# Current Insights into Tissue Injury of Giant Cell Arteritis: From Acute Inflammatory Responses towards Inappropriate Tissue Remodeling

**DOI:** 10.3390/cells13050430

**Published:** 2024-02-29

**Authors:** Dimitris Anastasios Palamidas, Loukas Chatzis, Maria Papadaki, Ilias Gissis, Konstantinos Kambas, Evangelos Andreakos, Andreas V. Goules, Athanasios G. Tzioufas

**Affiliations:** 1 Department of Pathophysiology, School of Medicine, National and Kapodistrian University of Athens, 11527 Athens, Greece; dpalamid@med.uoa.gr (D.A.P.); lukechatzis@gmail.com (L.C.); agoules@med.uoa.gr (A.V.G.); 2 Laboratory of Immunobiology, Center for Clinical, Experimental Surgery and Translational Research, Biomedical Research Foundation of the Academy of Athens, 11527 Athens, Greece; mpapadaki@bioacademy.gr (M.P.); vandreakos@bioacademy.gr (E.A.); 3 Department of Thoracic and Cardiovascular Surgery, Evangelismos General Hospital, 11473 Athens, Greece; gissisilias@hotmail.com; 4 Laboratory of Molecular Genetics, Department of Immunology, Hellenic Pasteur Institute, 11521 Athens, Greece; kkampas@hotmail.com; 5 Research Institute for Systemic Autoimmune Diseases, 11527 Athens, Greece

**Keywords:** giant cell arteritis, pathogenetic mechanism, monocytes, senescence, tissue remodeling, tissue injury

## Abstract

Giant cell arteritis (GCA) is an autoimmune disease affecting large vessels in patients over 50 years old. It is an exemplary model of a classic inflammatory disorder with IL-6 playing the leading role. The main comorbidities that may appear acutely or chronically are vascular occlusion leading to blindness and thoracic aorta aneurysm formation, respectively. The tissue inflammatory bulk is expressed as acute or chronic delayed-type hypersensitivity reactions, the latter being apparent by giant cell formation. The activated monocytes/macrophages are associated with pronounced Th1 and Th17 responses. B-cells and neutrophils also participate in the inflammatory lesion. However, the exact order of appearance and mechanistic interactions between cells are hindered by the lack of cellular and molecular information from early disease stages and accurate experimental models. Recently, senescent cells and neutrophil extracellular traps have been described in tissue lesions. These structures can remain in tissues for a prolonged period, potentially favoring inflammatory responses and tissue remodeling. In this review, current advances in GCA pathogenesis are discussed in different inflammatory phases. Through the description of these—often overlapping—phases, cells, molecules, and small lipid mediators with pathogenetic potential are described.

## 1. Introduction

**Figure 1:**
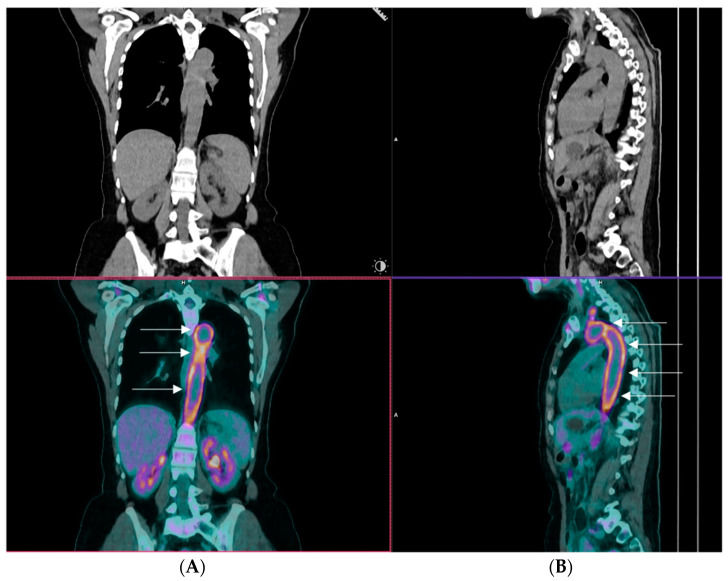
A representative image of an 18F-fluorodeoxyglucose positron emission tomography/computed tomography (PET/CT) of a patient with giant cell arteritis and extracranial large vessel involvement. The image displays coronal (Panel (**A**)) and sagittal views (Panel (**B**)), highlighting robust tracer uptake within the arch of the aorta, which is shown by arrows (kindly provided by our collaborator Dr CD Anagnostopoulos (BRFAA)).

Giant cell arteritis (GCA) stands out as the most prevalent form of primary systemic vasculitis in the elderly [1]. It is an autoimmune granulomatous disease that can affect large arteries with a notable predilection of the aortic arch and its branches. The superficial cranial branches of the external carotids, along with the ophthalmic branch of the internal carotid artery, are typically involved. Despite historical recognition of the systemic repercussions, manifested through constitutional symptoms and elevated inflammatory marker levels, GCA has traditionally been considered a predominantly cranial disease [2]. However, applying advanced imaging techniques has unveiled a broader spectrum, extending beyond cranial vasculature to involve other extracranial large and medium-sized vessels, underscoring the inherently systemic nature of GCA at the tissue level [3] (Figure 1). GCA is often associated with polymyalgia rheumatica (PMR), characterized by abrupt-onset pain, aching, and morning stiffness in the shoulder and hip girdle muscles. PMR is also an independent disease, suggesting that these two disorders could represent distinct expressions of a common underlying pathological process [4].

### 1.1. Epidemiology

Despite its global prevalence, GCA exhibits geographic variability in its incidence and prevalence. Regardless of the diagnostic criteria used, several studies consistently identify people of northern European ancestry with the highest reported incidence (44 cases per 100,000 persons aged more than 50 years) and Asians, particularly in southeast Asia, as the area with the lowest (0.4 per 100,000 persons aged 50 years and above) [5]. Notably, there is a discernible north-to-south decreasing gradient even across Europe, highlighting the potential influence of ethnic origins in disease development. Females exhibit an almost threefold increased likelihood of developing GCA compared to males, with a propensity for more extracranial artery involvement and a higher prevalence of PMR [6]. Seasonal variation adds another layer to the epidemiological profile, with a notable preponderance of diagnoses during the spring and summer [7,8].

### 1.2. Risk Factors

Among the various risk factors associated with the development of GCA, age emerges as the single most important determinant. GCA abstains from affecting individuals under the age of 50, and after that, its incidence steadily ascents, with more than 80 percent of diagnosed patients surpassing the age of 70 [9]. This age-dependent pattern highlights the intricate interplay between aging and the pathogenesis of the disease. The etiology hangs between the age-related restructuring of the immune system and the age-related vascular remodeling and dysfunction. Immunosenescence, characterized by the reduction of the naïve T-cell pool, contraction of T-cell diversity, and accumulation of memory T-cells, is accompanied by low-grade inflammation, commonly referred to as inflammaging [10]. Vascular aging introduces pivotal changes in the structural properties of the vascular wall, leading to increased arterial stiffness and reduced compliance [11]. The interplay between these two aging-related phenomena forms a complex network that contributes to the initiation of GCA. Unraveling the most prominent driving forces in the aging process remains a critical challenge for a better understanding of the pathophysiology of GCA.

### 1.3. Clinical Picture

Regardless of the underlying pathogenetic origins, GCA presents with a variety of clinical features extending from constitutional symptoms to headaches, scalp tenderness, jaw claudication, and visual manifestations [12]. The onset of the disease typically follows a subacute trajectory over several weeks, although abrupt presentations are not uncommon. If left untreated, GCA poses the ominous risk of its most dreaded complication, permanent visual loss and, less frequently, stroke. Notably, recent reports indicate a decline in the incidence of visual loss, dwindling from 20% of patients to less than 10%, attributed to the advancements in awareness, timely diagnosis facilitated by rapid temporal artery color Doppler ultrasonography assessment [13], and swift medical intervention [14]. Although visual loss can occur abruptly and without warning, single or multiple episodes of transient visual loss (amaurosis fungax) may precede [15]. These brief episodes, though temporary, serve as critical warning signs, offering a window of opportunity for prompt recognition and initiation of treatment. According to the latest recommendations from EULAR in 2023, ultrasound, including assessment of the axillary arteries, is now advocated as the first-line imaging diagnostic test for GCA. If there is a high clinical suspicion coupled with a positive imaging test, a biopsy can be omitted from the diagnostic procedure [16]. However, clinical suspicion should always be heightened for GCA mimickers, with the most prominent being ANCA vasculitis, amyloidosis, and atherosclerosis [17]. Lately, COVID-19 has also been added to the list of conditions with similar presenting symptoms, such as headaches and acute phase responses. Thrombocytosis may lean more towards GCA, while lymphopenia may suggest COVID-19, aiding in the correct diagnosis [18].

### 1.4. Treatment

The current management of GCA faces challenges typified by suboptimal rates of maintaining remission. Glucocorticoids (GCs) serve as the cornerstone of GCA treatment employed for both initiation and maintenance therapy in the majority of cases. While GCs effectively inhibit the Th17-dependent pathway, their impact on the Th-1-dependent pathway—crucial for ischemic complications and long-term arterial sequelae—is limited [19]. Notably, when the GC dose is tapered below 15–20 mg/day, relapses occur in approximately 50% of patients. To address this, the incorporation of conventional synthetic disease-modifying anti-rheumatic drugs (csDMARDs) becomes necessary. However, the recent approval of tocilizumab, an anti-interleukin 6 (IL-6) receptor inhibitor, provided new treatment options, especially for relapsing and refractory disease [20]. Several other agents, including sarilumab (a monoclonal antibody against IL-6Rα), ustekinumab (a monoclonal antibody targeting both IL-12 and IL-23), mavrilimumab (a human monoclonal antibody inhibiting the GM-CSF receptor), and baricitinib (inhibitor of JAK1 and JAK2 enzymes), are currently undergoing clinical investigation [21].

### 1.5. Histopathology

**Figure 2:**
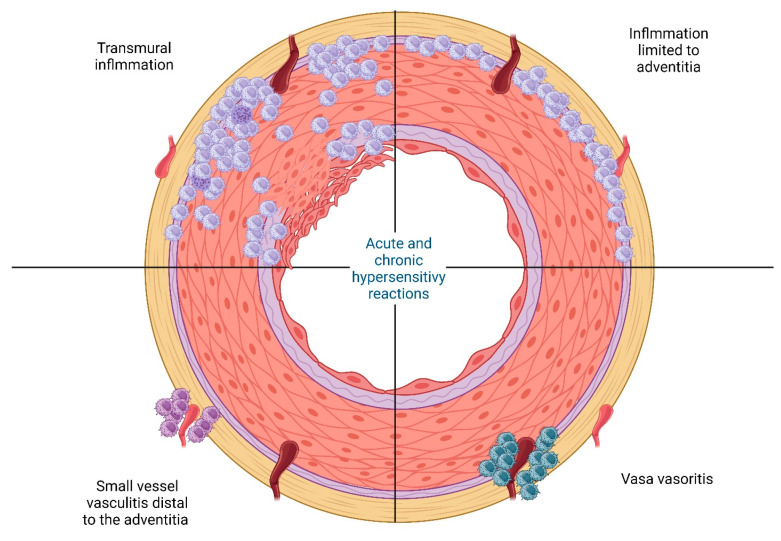
A visual representation showing the four different histological patterns observed in giant cell arteritis. Upper left: transmural involvement; upper right: Inflammation limited to adventitia; lower left: small vessel vasculitis; and lower right: inflammation limited to vasa vasorum [22]. Created with BioRender.com (accessed on 24 December 2023).

Similar to the diversity of clinical phenotypes, a remarkable heterogeneity also exists in the histopathologic pattern of the disease, delineated into four distinct categories: (i) transmural inflammatory pattern, (ii) inflammation confined to the adventitia, (iii) small vessel vasculitis distal to the adventitia, and (iv) vasa vasoritis [22] (Figure 2).

GCA is considered the prototype autoimmune disease where both acute and chronic delayed-type hypersensitivity reactions (DTH) may unfold. Beyond the chronic granuloma lesion, which stands as the hallmark of the disease, various subsets of immune cells, cytokines, and other molecules priming the autoimmune response have been described. However, the wealth of knowledge is vastly limited at single time points, and these are often compartmentalized, posing challenges in the synthesis and interpretation of such data. In this review, we shall discuss recent advances in understanding the pathogenetic mechanisms of GCA. The findings will be structured according to the disease’s different stages, including the initiation, perpetuation, and sustainability of the inflammatory response and the operative mechanisms also involving stromal cells and, eventually, leading to tissue injury. While acknowledging the overlap inherent to many of these phases, this organized presentation seeks to provide a comprehensive and accessible framework to navigate the intricate landscape of GCA pathogenesis.

## 2. Pathogenetic Mechanism in GCA

### 2.1. Initiation Phase

Similar to almost all systemic autoimmune and autoinflammatory disorders, the triggering factor(s) of GCA remains unknown. Several reports in the past have tried to elucidate the role of infections. Furthermore, tissue alterations via intrinsic mechanisms such as cell stress and accelerated atherosclerosis have also been proposed as initiating disease events. The lack of spontaneous experimental models, along with the poor understanding of tissue injury in GCA at the very early stages of the disease, are serious obstacles to understanding the initial mechanistic events. The initiating elements, when present, act synergistically upon a particular gene profile of patients with GCA that extends from antigen presentation (MHCs), adaptive and innate immunity genes (e.g., IL-2, IFN-γ), and several regulatory genes (e.g., TLRs).

#### 2.1.1. Genetics

The genetic component has been considered an essential factor in GCA pathogenesis, based mainly on two observations: (i) reports on familial clustering of the disease [23,24,25] and (ii) an increased prevalence in Europeans of northern and Scandinavian ancestry [26]. With the utilization of molecular techniques from different scientific groups, they sought to decipher the genetic landscape of the disease. In the past two decades, genetic studies have been categorized into two types, according to the candidate-tested genes: (i) studies of targeted gene polymorphisms associated with disease susceptibility (Table 1) and (ii) genome-wide association studies.

In the first category, the main focus of research has included genes encoding the human leukocyte antigen (HLA) classes I and II and different molecules participating in the inflammatory response of GCA (IL-1β, IL-6, IL-10, IL-17A, IL-21, IL-23R, IL-33, TNF-α, VEGF, MMP-9, MPO, and others). Several studies in small patient cohorts interrogated the role of the HLA region in the disease (Table 1). They revealed an association of GCA with the expression of HLA-DRB1*04 alleles (HLA-DRB1*0401, HLADRB1*0404, and HLADRB1*0408 haplotypes) [27,28,29,30,31,32,33,34,35,36]. Studies of genes encoding inflammatory molecules yielded various results. The rs2250889 polymorphism of the MMP-9 gene was associated with GCA susceptibility, but the study included only 30 patients [37]. Salvarani et al. demonstrated an association of the G/R 241 SNP in the ICAM-1 gene with the disease [38], but Amoli et al. reported a lack of association [39]. These studies included several weaknesses in sample size, lack of successful replicability, absence of appropriate geographical sample representation, and the use of low throughput technologies that did not allow for a large number of tested SNPs (Table 1) [40].

The research in this field entered the high-throughput screening era, producing more interesting results for the community. A large-scale study revealed that the HLA class II region is strongly associated with GCA [41], differentiating GCA from other systemic vasculitides that are genetically linked to HLA class I molecules (Takayasu arteritis, Behçet’s disease) [42] and supporting the implication of antigenic presentation processes in the initiation phase of the disease. A genome-wide association study (GWAS) confirmed this association, in addition to the identification of the plasminogen (PLG) gene polymorphism rs4252134 and the Prolyl 4-hydroxylase subunit alpha-2 (P4HA2) gene polymorphism rs128738 as genetic risk factors in GCA [43]. Furthermore, a meta-analysis highlighted the IL12B gene polymorphism rs755374 as a new risk SNP for GCA and a re-verification of the strongly associated HLA class II genes [44].

Overall, different studies exploring the contribution of SNPs in genes encoding proteins associated with the proinflammatory and tissue remodeling phases of GCA suggested the relevance of a plethora of innate and adaptive immunity molecules to disease pathogenesis. Additionally, GWAS introduced new genes as risk factors in GCA and reinforced the hypothesis of an unknown antigen-driven immune response initiating the pathogenesis of the disease.

**Table 1:** Genetic studies in GCA focused on genes encoding MHC class II and various inflammatory molecules.

Genes and No. of SNPs Tested	GCA Patients/ Controls	Associated SNP/Haplotype/ Variant with GCA *	Method	Ref, Year
HLA DRB1 alleles	20/242	-	MLCA	[33], 1991
HLA DRB1 alleles	41/384	HLA DRB1*04 (<0.001)	PCR	[31], 1998
HLA DRB1 alleles	42/63	HLA DRB1*0401 or B1*0404/8 (<0.03)	PCR	[34], 1992
HLA DRB1 alleles	65/200	HLA DR4 (<0.05)	MLCA	[27], 1988
HLA DRB1 alleles	42/1609	HLA DRB1*04 (0.0005)	PCR	[28], 1998
HLA DRB1 alleles	52/72	HLA DRB1*04 (0.0001)	PCR	[35], 1994
MBL, HLA DRB1 alleles	65/193	HLA DRB1*04 (0.01)	PCR	[29], 2002
HLA DRB1 alleles	53/145	HLA DRB1* 0401 (<0.05)	PCR	[36], 1998
HLA DRB1 alleles	44/99	HLA-DRB1* 0401 HLA (0.02) and -DRB1* 0404 (0.04)	PCR	[30], 2004
CCR5: 1 SNP	176/180	-	PCR-RFLP	[45], 2013
CD24: 1 SNP	120/195	rs3838646 (0.01) and rs8743 (0.001)	Real-Time PCR	[46], 2008
CRH: 2 SNPs	62/147	-	PCR-RFLP	[47], 2002
CRP: 4 SNPs	125/234	-	Real-Time PCR	[48], 2009
eNOS: 3 SNPs	57/117	C/1/T (0.04) and C/1/G (0.02) haplotypes	PCR	[49], 2003
eNOS gene: 2 SNPs	91/133	894 G/T (0.003)	PCR-RFLP	[50], 2003
FCGR2A, FCGR3A, FCGR3B and FCGR2B: biallelic polymorphisms	85/132	FCGR2A-FCGR3A 131R-158F haplotype (0.01)	SSCP Assay and Sanger Sequencing	[51], 2006
ICAM-1: 2 SNPs	121/228	G/R 241 (0.00005)	PCR-RFLP	[38], 2000
ICAM-1: 2 SNPs	58/129	-	PCR-RFLP	[39], 2001
IFN-γ microsatellite	50/129	-	PCR	[52], 2004
IFN-γ: 4 SNPs and IL-4: 5 SNPs		IFN-γ: SNP2 rs2227284 (0.0001) and IL-4 haplotype: T-T-C-A-C (0.02)	Real-Time PCR	[53], 2004
IL-6: 1 SNP in the promoter	62/124	−174 G/C (0.03)	PCR	[54], 2002
IL-6: 1 SNP in the promoter	126/112	-	PCR	[55], 2005
IL-10: 2 SNPs in the promoter	140/200	IL-10 promoter: −592 C/A (0.004)	PCR-RFLP	[56], 2006
IL-10: 2 SNPs in the promoter	103/226	IL-10 promoter: −592 C/A (0.004)	Real-Time PCR	[57], 2007
IL17A: 5 SNPs	1266/3779	rs2275913 (0.01) and rs7747909 (0.04)	Real-Time PCR	[58], 2014
IL-18: 3 SNPs	212/405	rs360719 (0.003) and rs1946518 (0.02)	Real-Time PCR	[59], 2010
IL2/21: 1 SNP	272/791	-	Real-Time PCR	[60], 2011
IL23R: 1 SNP and IL12RB2: 1 SNP	357/574	IL12RB2: rs3790567 (0.039)	Real-Time PCR	[61], 2011
IL33: 6 SNPs and IL1RL1: 3 SNPs	1363/3908	IL33 gene: rs7025417 (0.041)	Real-Time PCR	[62], 2014
IRAK1: 1 SNP and MECP2: 1 SNP	627/1520 (only females)	-	Real-Time PCR	[63], 2014
IRF-5: 1 SNP and 1 indel	220/520	-	Real-Time PCR and Sanger Sequencing	[64], 2010
MCP-1: 1 SNP in the promoter	79/99	haplotypes C-C (0.03) and T-T (0.005)	Real-Time PCR	[65], 2005
MICA-TM microsatellite polymorphism and HLA-B genotyping	98/225	MICA A5 (0.0005) and HLA-B*15 alleles (0.04)	PCR	[66], 2007
MIF: 1 SNP in the promoter	83/122	-	Sanger Sequencing	[67], 2005
MMP-9: 7 SNPs	30/23	SNP rs2250889 (0.0009)	PCR-RFLP	[37], 2008
MPO: 1 SNP in the promoter	156/235	−463 G/A (0.0002)	PCR-RFLP	[68], 2008
NFKB1: 1 SNP in the promoter	96/204	-	Real-Time PCR	[69], 2006
NLRP1: 1 SNP	685/2898	rs8182352 (0.0026)	Real-Time PCR	[70], 2013
PTPN22: 1 SNP	99/229	-	Real-Time PCR	[71], 2005
PTPN22: 2 SNPs and CSK: 2 Variants	911/8136	PTPN22: rs2476601/R620W (0.00002)	Real-Time PCR	[72], 2013
STAT4: 1 SNP	212/678	-	Real-Time PCR	[73], 2009
TLR4: 1 SNP	210/678	SNP rs4986790 (0.01)	Real-Time PCR	[74], 2009
TNF microsatellite markers a-d	62/147	TNFa2 allele (0.003) and TNFa10 allele (0.02)	Sanger Sequencing	[75], 2000
TNF-α, IL-1A and IL-1B genes: 5 SNPs	57/128	-	PCR	[76], 2002
TRAF1: 1 SNP and C5: 1 SNP	220/410	-	Real-Time PCR	[77], 2010
VEGF: 3 SNPS in the promoter	92/200	I (0.025) and C634 (0.015)	PCR-RFLP	[78], 2003
VEGF: 2 SNPs	103/226	-	Real-Time PCR	[79], 2005
CCL2, CCR7, IL10, IL12A, IL1A, IL1B, IL1RN, IL6, IL8, IFNG, LTA, eNOS, TNF and VEGF: 130 SNPs	82/166	eNOS (rs2779251), VEGF (rs1885657, rs2010963, rs699946 and rs699947), IL1RN (rs17207494), IL-6 (rs7805828 and rs1546766) and CCL2 (rs1860190) SNPs (<0.05)	Illumina Bead Array System	[80], 2012

CCL2: C motif chemokine ligand 2; CCR5: C-C chemokine receptor 5 gene; CCR7: C-C chemokine receptor type 7; CD24: cluster of differentiation 24; CRH: corticotropin-releasing hormone; CRP: C-reactive protein; CSK: C-terminal Src kinase; eNOS: endothelial nitric oxide synthase; FCGR2A: Fc gamma receptor 2A; ICAM-1: intercellular adhesion molecule 1; IFN-γ: interferon gamma; IL: interleukin; IL1RL1: interleukin 1 receptor-like 1; IL12RB2: interleukin 12 receptor subunit beta 2; IL23R: interleukin 23 receptor; indel: insertion/deletion; IRAK1: interleukin-1 receptor-associated kinase 1; IRF-5: interferon regulatory factor 5; LTA: lymphotoxin-α; MBL gene: mannose-binding lectin gene; MCP-1: monocyte chemoattractant protein 1; MECP2: methyl CpG binding protein 2; MLCA: microlympocytotoxicity assay; MICA-TM: major histocompatibility complex class I chain-related gene A transmembrane; MIF: macrophage migration inhibitory factor; MMP-9: matrix metalloproteinase 9; MPO: myeloperoxidase; NLRP1: NACHT; LRR; FIIND; CARD domain and PYD domains-containing protein 1; PCR: polymerase chain reaction; PTPN22: protein tyrosine phosphatase non-receptor type 22; RFLP: restriction fragment length polymorphism; SNP: single-nucleotide polymorphism; SSCP: single-stranded conformational polymorphism; STAT4: signal transducer and activator of transcription 4; TLR4: toll-like receptor 4; TNF: tumor necrosis factor; TRAF1: tumor necrosis factor receptor-associated factor 1; VEGF: vascular endothelial growth factor. * *p*-values are in parentheses.

#### 2.1.2. Infections and Microbiome

The antigen-driven hypothesis of triggering the initiation of vascular inflammation observed in GCA has been a matter of debate for many years in the literature. In this direction, several studies have investigated the implication of different infectious agents in vascular dendritic cell activation and subsequent disease onset [81]. Of the infectious agents interrogated, human herpes virus (HHV)-6, HHV-7, varicella-zoster virus, and Epstein–Barr virus have not been associated with GCA. Although cytomegalovirus, parvovirus B19, herpes simplex virus, human parainfluenza 1, and chlamydia pneumonia were initially correlated with the disease, none of these results were confirmed by subsequent studies in larger cohorts [81]. A previous study reported the presence of varicella-zoster virus (VZV) antigen in 64–73% of temporal artery biopsies (TABs) of large vessel vasculitis (LVV) patients, with or without temporal artery involvement, as opposed to 22% of normal arteries tested [82]. Although this finding implied a possible disease-causing mechanism of VZV infection, the study lost its validity after Pisapia et al. reported an increased false-positive rate due to non-specific staining of the antibody used in the immunohistochemistry assay for VZV detection [83].

State-of-the-art approaches such as whole genome sequencing and 16S Ribosomal RNA gene sequencing have also been utilized in search of infectious agents implicated in GCA pathogenesis. In a shotgun sequencing-based study, none of the previously reported pathogens were detected in GCA TABs. Moreover, the microbiome did not differ between GCA cases and controls since only members of the normal skin flora were observed [84]. Two studies of another group that followed aseptic techniques during tissue collection found that the microbiome of thoracic aortic aneurysms from isolated aortitis and GCA differ substantially from the microbiome of non-inflammatory aortic aneurysms [85] and that from TABs of GCA patients [86]. These studies provided novel insights into the candidate role of the microbiome of different vascular components in health and disease. However, the translation of these findings in a comprehensive manner that could enrich our knowledge of GCA pathogenesis still has a long way to go.

#### 2.1.3. Vascular Aging/Inflammaging

Undoubtedly, increasing age is a major etiological factor in GCA’s initiation, with different compartments of immunity being affected [11]. T-cells, in particular, exhibit a senescent phenotype due to the loss of the CD28 cell surface marker [87]. CD4+ CD28− and CD8+ CD28− cells are reported to be enriched in T-cell subsets of aged individuals [88]. There is also a marked decrease in the numbers of naïve T-cells and the T-Cell Receptor (TCR) repertoire diversity as opposed to an increase in T effector and memory cells [89]. Dendritic cells (DCs) can also be impacted by aging, displaying impaired activation and migration capacity [90]. Senescent macrophages display an altered, more inflammatory phenotype characterized by the expression of proinflammatory mediators (IL-1β, IL-6, TNF-α) [91]. It is reported that epigenetics may play a vital role during this so-called inflammaging process. Indeed, DNA methyltransferase 1 (DNMT1) levels in T-cells are decreased during the aging process [92]. DNMT1 is an enzyme that facilitates the maintenance of DNA methylation levels between cell divisions, a disturbed process during aging that leads to altered methylation levels in T-cells, a phenomenon associated with increased risk for autoimmunity and cancer. On the other hand, structural arterial changes are observed that correlate with increasing age, including calcium deposition, increased artery stiffness, wall thickening, and alterations in the extracellular matrix of the arteries [93]. The effect of inflammaging on the immune system, which is disturbed in GCA, and the vasculature, the target organ of the disease, supports the central role of senescence in GCA pathogenesis.

### 2.2. Perpetuation and Sustainability of the Inflammatory Response

Different studies of tissue biopsies of GCA patients reveal many types of immunocytes involved in innate and adaptive immune responses that are usually activated as attested by cellular markers and cytokines. Essentially, the perpetuation of tissue inflammatory response is performed through acute DTH mechanisms in which activated monocytes play a central role. At the same time, sustainability is implemented through chronic DTH responses with granuloma formation, which is the histological hallmark of the disease. In some instances, the local autoimmune injury is probably continuously fueled by the formation of ectopic germinal centers [94]. Disturbances in immune cell subpopulation frequencies in peripheral blood mononuclear cells (PBMCs) are also observed during active and inactive disease states [95]. An unmet need of GCA is the exact definition of cells participating in the acute inflammatory response in correlation with the cells in remission. Indeed, the type of cells and the inflammatory mediators differ in the acute and remission phases since the metabolic landscape is also different [96]. To this end, other groups and ours have suggested the investigation of pairs of biological samples in both activity and remission states in an attempt to gain clinically relevant biomarkers. Hereafter, the types and functions of major immune cell types are described.

#### 2.2.1. Dendritic Cells and TLRs

The activation of resident DCs and subsequent activation of T-cells in the affected GCA arteries is thought to be the initial step of the intense inflammatory response. Following sensing intrinsic or extrinsic antigens, these cells induce strong innate and adaptive immune responses via TLR ligation and cell activation, which leads to chemokine and cytokine production, orchestrating tissue infiltration from T-cells. Thus, TLR differential expression and activation in the context of this pathology is of great importance [97].

Resident DCs observed in the adventitia of normal arteries display an immature phenotype [98,99] and contribute to immune surveillance. Detection of activated DCs in TABs is not exclusive to GCA but has also been described in TABs of patients with PMR. Activated DCs are located in the adventitia of PMR patient TABs as opposed to GCA patient TABs, in which activated DCs are extended in all three layers of the vascular wall [99]. Moreover, DCs are restricted to the artery and do not migrate to lymph nodes [98], a phenomenon facilitated by a positive-feedback loop of specific chemokine production (CCL19 and CCL21) and their receptor, CCR7 [99], leading to the migration of more DCs at the site of inflammation and restricting their escape to the lymph nodes. Additionally, DCs express CD83, an activation marker, and CD86, a co-stimulatory molecule that renders them capable of activating naïve T-cells. A study in a human artery–mouse chimera model of GCA showed that DC depletion with an antibody against CD83 was able to effectively reduce the inflammatory response, T-cell infiltration, and IFN-γ production [99], recapitulating the significance of DCs in the initial inflammatory cascade of GCA. It has also been demonstrated that the immunoprotective PD-1/PD-L1 immune checkpoint is defective in GCA-affected tissue DCs, thus rendering them more susceptible to activation [100]. Interestingly, a study investigating the expression profile of TLR genes of 6 GCA-affected arteries obtained during autopsy showed a distinct TLR profile for each affected vessel studied [101]. Until recently, there were no reports on DC subpopulations in the peripheral blood of GCA patients. Reitsema et al. investigated the frequencies of DC subsets in the peripheral blood of GCA and PMR patients compared to healthy individuals for the first time [102]. The authors noted that plasmacytoid DCs (pDCs) and type-2 conventional dendritic cells (cDC2) frequencies did not differ between patients and controls, whereas cDC1 frequencies were substantially reduced in patients. Additionally, in GCA/PMR patients, pDCs exhibited higher expression of the immune checkpoint CD86 and reduced expression of CD40, whereas cDC2 subsets exhibited lower activation status as shown by lower HLA-DR activation marker expression [102]. Overall, the interpretation of these results is challenging in understanding GCA pathogenesis since there are no surrogate data of DC subsets on the inflamed tissue level.

Examining the mechanism of DC activation in the context of the disease is pivotal in understanding the clinical heterogeneity in GCA, with some patients exhibiting disease restricted to temporal arteries while others display a more pronounced inflammatory response in different large vessels. A multidimensional study correlating TLR repertoire, pathogen expression (bacteria and viruses), and the activation status of different DC subpopulations in different anatomically affected arteries has never been conducted and may provide insights into understanding the etiology and initiation phase of GCA.

#### 2.2.2. CD4 T-Cells

In GCA, a pronounced T-cell compartment participates in the underlying inflammation [103]. Indeed, activated DCs recruit CD4+ T-cells at the site of inflammation through CCL18, CCL19, CCL20, and CCL21 chemokine expression [104]. Upon arrival, activated CD4+ cells differentiate into Th1 and Th17 T-cell subsets through proinflammatory signals of the inflamed artery (IL-1β, IL-6, IL-12, IL-18, and IL23) [105,106]. In Th1 responses, CD4+ cells, under the influence of IL-12 and IL-18, differentiate into Th1 cells that secrete IFN-γ [107], a critical regulatory cytokine in the disease’s pathogenesis [108]. On the other hand, Th17 responses include the differentiation of CD4+ cells into Th17 cells under the effect of IL-1β, IL-6, and IL23 [107]. It is well established that these two T-cell axes operate in the vicious, inflammatory cycle observed in the disease. However, the order of appearance of Th1 and Th17 responses is unknown. GC treatment is highly effective in restricting Th17 responses, as proved by the reduction of both the number of Th17 cells in the peripheral blood and IL-17 serum levels [105,109]. However, IFN-γ producing Th1 responses show resistance after GC treatment in GCA patients [105,110].

Another dysregulated CD4+ subset implicated in GCA pathogenesis is T regulatory cells (Tregs). Tregs are reduced in both peripheral blood and at the tissue level in GCA [105,106,111]. Moreover, the high levels of IL-6, IL-21, and IL-23 in the inflamed tissue microenvironment lead to the restricted expression of FOXP3 transcriptional factor, which is crucial for the differentiation of Tregs, favoring the upregulation of RORγt transcriptional factor that mediates Th17 differentiation [112]. An imbalance of the Th17/Treg axis participating in the GCA pathogenetic mechanism has been observed and is restored by IL-6R blockade as shown by normalization in Treg peripheral blood frequencies and a decrease in activation status [111,113]. Miyabe et al. also highlighted that Tregs in GCA present a reduced suppressor capacity, possessing a proinflammatory phenotype with increased production of IL-17 [114].

Recent evidence has emerged for the role of Th9 cells in GCA. Th9 cells constitute a distinct lineage of T-cells differentiated from CD4+ naïve cells under the influence of IL-4, TGF-β, and thymic stromal lymphopoietin (TSLP) and are effective producers of IL-9. In GCA, Th9 cells are mainly observed in artery tissues with transmural inflammation and small vessel vasculitis, as opposed to their absence in vasa vasorum vasculitis [115]. Furthermore, patients with GCA exhibit a defective, macrophage-induced CD155-CD96 immune checkpoint that leads to Th9 expansion [116]. The same study showed increased vessel wall destruction due to increased IL-9 production in a humanized mouse model of GCA [116]. Although all groups have not demonstrated the functions and disease relevance of the Th9 subset in GCA, their implication in the pathogenic phenomena appears important and requires further research.

GCA-affected tissue may possess ectopic germinal center (EGC)-like structures that are important for perpetuating local inflammatory responses. In the formation of such structures, Follicular helper T (Tfh) cells play a central role. Tfh cells have been detected in the inflamed artery and the peripheral blood of GCA patients [117]. Tfh cells are capable of producing IL-21, which, among other functions, increases B-cell differentiation into plasmablasts and triggers germinal centers to produce immunoglobulins [118]. However, autoantibodies characterizing the disease have not been detected yet.

#### 2.2.3. CD8 T-Cells

For years, the role of CD8+ T-cells in GCA has not attracted much attention due to their low number in both the periphery and tissue [119,120,121]. Nonetheless, CD8+ cells present oligoclonality in GCA tissue. They may infiltrate temporal arteries, producing proinflammatory cytokines such as IL-17A and IFN-γ as well as cytotoxic substances such as granzymes A and B [119]. These observations give rise, once again, to an antigen-driven theory of GCA initiation. Furthermore, a decreased number of immunosuppressive CD8+ CCR7+ FoxP3+ Tregs has been observed in the peripheral blood of GCA patients, potentially leading to a failure in the control of CD4+ T-cell proliferation and activation through a NADPH oxidase-2 dependent mechanism [122]. In addition, a decrease in CD8+ T-cells was associated with aging and GCA pathogenesis [122]. The fact that this defect was not abrogated by GC treatment in patients raises new questions about the pathogenetic role of CD8+ cells.

#### 2.2.4. B-Cells

The role of B-cells in GCA had been overlooked for many years due to the initial reports of a low number of B-cells infiltrating the temporal artery [123,124], suggesting that humoral immunity plays a marginal role in the inflammatory process. Data on serum autoantibodies in GCA patients support a more active role for B-cells in the inflammatory process. Autoantibodies that were identified include (i) low titers of anti-cardiolipin of the IgG isotype that disappeared following GCs treatment [125,126,127,128]; (ii) anti-endothelial cell antibodies which were not specific for GCA and were also detected in other systemic vasculitides [129,130]; and (iii) anti-smooth muscle cell antibodies, but the study included a low number of patients [130]; additionally, (iv) Baerlecken et al. reported antibodies against the human ferritin heavy chain in a high percentage of GCA patients (92%) [131] while Régent et al. reported lower titers, but yet with high prevalence (71.9%) in GCA patients and 34% in patients with a diagnosis other than GCA diagnosis [132]. These results collectively support the idea that these autoantibodies are not disease-specific but an epiphenomenon of the inflammatory bulk.

In recent years, the formation of tertiary lymphoid organs (ATLOs) has been identified in the aortas of GCA patients but not in the affected temporal arteries [94,133]. Moreover, those ATLOs were found in the adventitia of inflamed aortas and were absent from GCA-positive temporal arteries, suggesting different organization according to the size of the affected artery. ATLOs consist of follicular dendritic cells located near T-cells and endothelial cells in the media layer, supporting the organization of lymphoid tissue against arterial wall-derived antigens. In the peripheral blood of treatment-naïve GCA and PMR patients, B effector cells are decreased compared to healthy controls and reach normal levels after GC treatment, exhibiting an enhanced IL-6 production capacity [134]. In contrast, serum levels of CXCL9 and CXCL13, major chemokines for the organization of ATLOs, are elevated [135,136]. The same study highlighted that B-cells were detected in the inflamed arterial tissue, and their migration pattern followed the CXCR3–CXCL9 and CXCR5–CXCL13 chemokine axes [136]. The above data indicate that B-cells in active disease are reduced in the peripheral blood due to their migration to the affected temporal arteries. In cases of large vessel involvement, ectopic lymphoid tissue is generated and is possibly connected to chronic inflammatory processes.

#### 2.2.5. Monocytes

The abundant expression of IFN-γ in the initial steps of inflammation induces CCL2 expression by vascular smooth muscle cells (VSMCs), leading to tissue infiltration by monocytes of the classical subset, expressing the CCR2 receptor [137]. In addition, non-classical monocytes also participate in the inflammatory process and reach out to the tissue lesion under stimuli of the CX3CR1–CX3CL1 axis [138]. The predominant type of monocytes in the GCA tissue lesions is still a matter of debate, with different groups reporting either the classical monocyte subset [137] or non-classical subsets as the major monocytic type [138]. In the peripheral blood of GCA patients during diagnosis, elevated classical monocyte numbers [95,138] persisted after three months of GC treatment. Interestingly, disease remission correlated with a decrease in the number of intermediate and non-classical monocytic lineages, indicating that GCs act partly towards normalizing monocyte numbers [138]. Following integrative analysis of the methylome and transcriptome of CD14+ cells in GCA active and inactive disease, a recent study identified IL-11 as a novel cytokine pathway associated with the disease [139]. However, functional studies on the relevance of their findings to disease pathogenesis are needed.

#### 2.2.6. Cytokines with a Significant Contribution to the Inflammatory Response of GCA

IL-6: IL-6 is considered to be the leading inflammatory cytokine in GCA’s pathogenetic mechanism. Serum levels of IL-6 appear to be elevated early during the disease course [105] and decrease rapidly after a few hours of GC initiation [140], without, however, reaching the levels of healthy individuals, even after chronic administration of GCs [141]. In addition, after GC tapering, IL-6 is among the first cytokines that arise in GCA patients’ serum [140]. IL-6 also correlates highly with acute phase reactants (erythrocyte sedimentation rate and C-reactive protein) and may serve as a biomarker of disease activity [142]. The above clinical observations made IL-6 a therapeutic target, and tocilizumab, a humanized monoclonal antibody to the IL-6 receptor (IL-6R), is now consistently used for patients with relapsing or refractory disease [143].

Although targeting of IL-6 is successful in therapy, the exact mechanism of action in GCA pathogenesis has not been elucidated, probably due to its pleiotropic effects. IL-6 is expressed by various immune and stromal cells, including activated monocytes, macrophages, B-cells, T-cells, fibroblasts, and endothelial cells [144]. IL-6 signal transduction is mediated by the complex IL-6/IL-6R/gp-130, with the latter being ubiquitously expressed, while hepatocytes, activated monocytes, macrophages, B-cells, and endothelial cells mainly express IL-6R. An established function of IL-6 is to control the balance between Th17 cells and Tregs [145], which is also evident in GCA [111,114]. The reduced frequency and the proinflammatory phenotype of Th17 expressing Tregs are reversed in GCA patients after tocilizumab treatment. There are conflicting reports on the effect of IL-6 on tissue remodeling. In a study by O’Neill et al., serum amyloid A (SAA) protein, which is triggered by IL-6 hepatocyte signaling and is increased in GCA patients’ serum, induced the protein expression of vascular endothelial growth factor (VEGF) and MMP-9 in an ex vivo culture model of temporal arteries. However, a more recent study of the same group argues a role of IL-6 in tissue remodeling since there was no effect of IL-6 treatment on myofibroblast proliferation and migration in a myofibroblast outgrowth culture model of GCA [146].

GM-CSF: Granulocyte-macrophage colony-stimulating factor (GM-CSF) has been recently suggested as a highly influential cytokine in GCA [147]. This proinflammatory cytokine is expressed by fibroblasts, endothelial, epithelial, myeloid, and T-cells upon appropriate stimulatory cues [148]. The GM-CSF heterodimeric receptor is composed of an α chain specific to GM-CSF and a signal transduction β chain, which is also found in the IL-3 and IL-5 receptors and, upon phosphorylation, triggers the activation of the JAK2/STAT5 signaling pathway [148]. Patient serum levels of GM-CSF are extremely low and comparable with healthy individuals [147]. On the contrary, GM-CSF protein levels are increased in GCA TABs [147] and PBMCs upon stimulation in vitro [106], suggesting a paracrine function of this cytokine on the inflamed tissue. The only functional study about GM-CSF in GCA was by Corbera-Bellalta et al. and highlighted the role of this cytokine in GCA. GM-CSF blockade by mavrilimumab, a fully human IgG4 monoclonal antibody to GM-CSFRα, abolished immune cell infiltration, inflammatory markers, and tissue remodeling factors, suggesting a role of GM-CSF in both the perpetuation of the inflammatory response and tissue injury phases [147]. A phase-2 clinical trial on mavrilimumab showed promising results on sustaining disease remission in week 26. However, further clinical trials are needed to determine whether this treatment modality is superior to current treatment options [149].

### 2.3. Tissue Injury

GCA is characterized by vital organ and life-threatening comorbidities as a result of ongoing tissue injury. The most severe and common are blindness and aneurysm formation. To this end, the mechanisms involved in tissue remodeling following acute inflammatory responses are of paramount importance. This section will discuss cells and molecules mediating tissue injury and their relation to acute inflammatory responses (MMPs, cytokines, and immune-mediated thrombosis).

#### 2.3.1. Neutrophils

Until recently, there was scarce information in the literature about the presence and involvement of neutrophils in GCA. A few reports suggested a low prevalence of neutrophil infiltration in GCA biopsies [22,150]. Our group has reported the presence of neutrophils in GCA TABs and identified their localization in the adventitia and media of inflamed arteries [151]. Interestingly, we identified the release of neutrophil extracellular traps (NETs) decorated with proinflammatory mediators (IL-6 and IL-17A) in the adventitia adjacent to the vasa vasorum. Another group identified a subset of immature neutrophils with a phenotype of CD66b+CD15+CD10lo/–CD64– in peripheral blood of GCA patients and granulomatosis with polyangiitis (GPA) patients, but not in healthy individuals [152]. This immature cell subset was resistant to apoptosis, exhibited high potential for generating reactive oxygen species (ROS), and infiltrated the inflamed tissue. Neutrophilia is observed in the peripheral blood of GCA treatment-naïve patients during active disease, [153]. Neutrophils possess three distinct phenotypes with GC treatment over 24 weeks [153]. Neutrophils in weeks 1 and 24 exhibit high capabilities for endothelial adhesion, and neutrophils in week one can suppress T-cell proliferation as opposed to week 24. Based on the loss of neutrophil subsets with a capacity to suppress T-cell responses during GC tapering, the authors suggested a possible role of neutrophils in disease relapse, but further research is needed to support this hypothesis. Serum biomarkers related to neutrophils have also been detected in GCA patients. These include calprotectin and N-formyl methionine (fMET), markers of neutrophil activation, which are increased in the serum of GCA [154], as well as higher levels of circulating NETs in LVV patients when compared to healthy controls [155]. However, the circulating levels of NET components are non-indicative of NET release [156] and these findings have not been validated in larger cohorts. Another unexplored role of NETs is their implication in immunothrombosis, which may also play a role in tissue injury observed in GCA since intralaminal thrombosis is observed in 10–20% of positive GCA biopsies. There have been reports of decorated NETs with tissue factor (TF) in active lupus kidney lesions. Moreover, in a mouse model of sepsis-induced acute lung injury, NET generation mediated by stimulator of interferon genes (STING) activation resulted in endothelial damage via enhanced production of TF. Taken together, these findings suggest further research on the role of neutrophils in promoting immunothrombosis in GCA.

Overall, neutrophil biology in GCA requires further exploration to understand their role in tissue remodeling and/or disease relapse.

#### 2.3.2. Macrophages

Macrophages are critical players in the inflammatory bulk identified in TABs of GCA patients. Therefore, they are thought to be of major importance both during the perpetuation of the inflammatory response and the subsequent vascular remodeling phase. Monocytes that reach the inflamed artery differentiate into macrophages and form multinucleated giant cells, a phenomenon that serves as the histological hallmark of the disease since giant cells are detected in 57–74.8% of GCA tissue biopsies [22,157]. The critical role of macrophages is manifested by producing several inflammatory mediators, including matrix metalloproteinases (MMPs) and growth factors, leading to tissue remodeling and vascular occlusion [158,159]. Macrophage subsets are heterogeneous, exhibiting high plasticity and various functions [160]; in GCA, research has focused on M1 and M2 macrophage subsets [161,162]. M1-like macrophages are located in the adventitia and media layers of the artery [163], secreting the proinflammatory cytokines IL-1β and IL-6, leading to the perpetuation of the inflammatory response [161]. MMPs that act on matrix degradation [117], reactive oxygen species (ROS) that disrupt the homeostasis of VSMCs, and endothelial cells (ECs) [163] have also been described. M2-like macrophages are distributed in the intima-media border of the artery and promote wound healing via the production of platelet-derived growth factor (PDGF) [164] and VEGF [165], forcing extensive tissue remodeling and vascular occlusion. Macrophage-mediated tissue destruction has been associated with CD206+/MMP-9+ macrophages, while FRβ+ macrophages are linked to intima proliferation, and their tissue distribution is regulated by local expression of GM-CSF and M-CSF, respectively [162]. The same group recently reported that GM-CSF skewed CD206+ macrophage production of the angiogenic factor YLK-40, which is also elevated in the serum of GC-treated patients. Moreover, the knockdown of YLK-40 resulted in a vast decrease in MMP-9 production from macrophages [166]. To this end, the authors propose that targeting the blockade of YLK-40 or GM-CSF could offer a therapeutic option in controlling inappropriate tissue remodeling, a still unmet need in the management of the disease.

Given the central role of macrophages in GCA pathogenesis and the high disease relapse rate after GC withdrawal, it is of great importance to study inflammasome activation pathways in the context of this disease. NLRP3 inflammasome hyperactivation has been associated with multiple autoimmune and autoinflammatory diseases, including Sjögren’s syndrome, SLE, ankylosing spondylitis, and RA [167], and has been proposed as a therapeutic target in managing chronic inflammation in these diseases. However, there are no studies interrogating the implication of inflammasome activation during perpetuation and sustainability of the inflammatory response in GCA.

**Figure 3:**
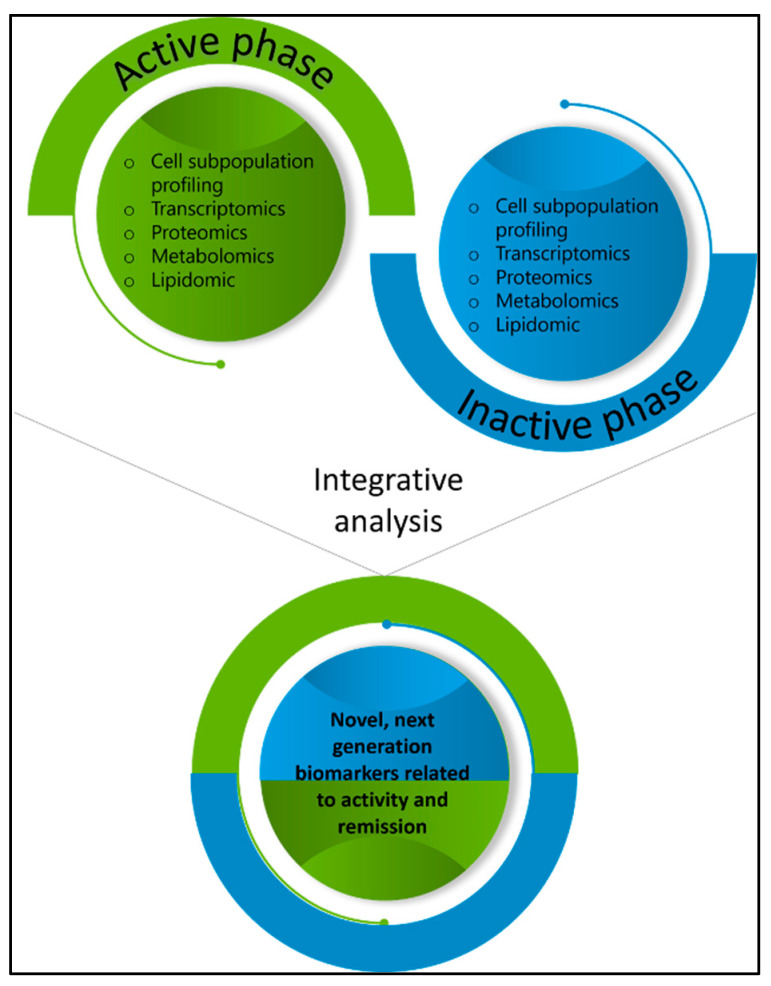
Proposal for the identification of novel next-generation biomarkers in GCA that possess two characteristics: (i) they are tightly related to the underlying pathogenetic mechanism; and (ii) they are the outcome of integrative analysis of high-throughput assays during disease activity and inactivity. They may also be potentially used as activity indices, organ damage markers, and treatment selection tools.

With the advancements in the field of novel PET/CT radiotracers and in an effort to implement precision medicine approaches in disease diagnosis and monitoring, several candidate radiotracers have been proposed for GCA imaging, including those targeting macrophages [168]. Although macrophages are highly relevant to disease pathogenesis during initiation and relapse, there is a limited number of studies investigating the role of this immune cell subset in GCA. We propose the implementation of a structured research strategy that involves the integrative analysis of different omics technologies in two-time points of the disease, activity and inactivity, to identify the most sensitive-to-change parameters that could serve as next-generation biomarkers to be utilized as radiotracers for non-invasive disease diagnosis and monitoring (Figure 3).

#### 2.3.3. Matrix Metalloproteinases (MMPs)

Macrophages, along with VSMCs, are the primary cellular source of MMP-2 and MMP-9 production, two enzymes thought to be involved in GCA tissue destruction via internal elastic lamina disruption [169]. There are several reports on the strong expression of MMP-9 in GCA lesions [162,170,171] as well as an increased ratio of MMP-9/TIMP1 [172], with the latter being a natural inhibitor of this gelatinase type. As mentioned before, MMP-9-mediated matrix degradation facilitates T-cell infiltration in the affected GCA arteries as described in a study utilizing a human artery-SCID-mouse model [117]. On the other hand, MMP-2 expression in GCA lesions is controversial since it is constitutively expressed by VSMCs and ECs, and its expression is comparable both in GCA tissue and non-GCA control arteries [169,173]. MMP-12 gene expression is highly upregulated in positive TABs and its expression is even more pronounced compared to MMP-9. However, its protein levels and enzymatic activity have not been evaluated in the affected tissue [174].

#### 2.3.4. Vascular Smooth Muscle Cells and Myofibroblasts

Previous studies have shown that VSMCs possess a critical role in the pathogenetic mechanism of GCA and, more specifically, in the perpetuation of the inflammatory response and the tissue remodeling stages [175]. Upon IFN-γ stimulation, VSMCs produce the chemokines CXCL9, CXCL10, CXCL11, and CCL2 that facilitate the recruitment of T-cells and monocytes at the site of inflammation. In an in vitro model of GCA, the blockade of IFN-γ resulted in the reduction of macrophage infiltration in cultured arteries due to reduced VSMC chemokine production [176]. In large arteries, VSMCs reside in the media layer and, upon stimulation, are capable of migrating into the intima, where they differentiate into myofibroblasts that proliferate, producing extracellular matrix proteins (collagen I, collagen III, and fibronectin) [177], leading to lumen stenosis and vessel occlusion [178]. The molecules that mediate the signaling cascade of VSMCs regarding wound healing are thought to be PDGF [164], TGF-β, and endothelin 1 (ET-1) [179,180]. ET-1 receptors A and B are expressed on the surface of VSMCs and their blockade reduces the proliferation and migration of VSMCs across the inflamed tissue [181]. Furthermore, imatinib mesylate treatment, which inhibits the PDGF receptor and is currently used in the treatment of lung fibrosis, reduced VSMC migration and proliferation in vitro, highlighting the central role of PDGF in mediating the activation of signaling pathways regulating the VSMC profile and possibly vascular occlusion in GCA [177]. Another element determining the fate of VSMCs during GCA is neurotrophin expression. In a study investigating their role in GCA, Ly KH et al. reported an enhanced expression of nerve growth factor (NGF), brain-derived neurotrophic factor (BDNF), tropomyosin receptor kinase B (TrkB), and sortilin in GCA temporal arteries compared to control arteries that also express these molecules to a lesser extent [182]. The authors also reported that NGF and BDNF facilitated VSMC migration and proliferation in vitro. However, these findings need further confirmation to elucidate whether neurotrophins have a primary or secondary role in vascular occlusion in GCA.

#### 2.3.5. Endothelial Cells

Endothelial cells (ECs) are observed as a thin layer surrounding the lumen of large vessels, but can also be found in the vasa vasorum of large arteries. Their primary function is to protect the inner walls of the artery from immune cell infiltration. However, during the inflammatory response in GCA, ECs are activated and express adhesion molecules (ICAM-1, ICAM-2, P-selectin, E-selectin, and VCAM-1) [183,184], thus facilitating T-cell adhesion and tissue infiltration. Some authors, among them our group, consider activation of the endothelium in vasa vasorum to be an initial finding resulting in the breach of tolerance in this immune-privileged region and spreading the inflammatory response in all layers of the artery, thus creating transmural inflammation, the most common histologic finding of GCA-positive biopsies. Vasa vasorum ECs, under the influence of serum VEGF, produce an enhanced expression of Jagged1, which is a Notch ligand [185]. The activation and upregulation of the Notch-mTORC1 pathway are currently considered the central module for CD4+ T-cell recruitment and polarization into Th1 and Th17 cells [186]. This mechanistic study is coupled to the already observed NOTCH1 upregulated expression in CD4+ cells of GCA patients [187].

## 3. Chronic Inflammatory Components on the Tissue Level

### 3.1. NETs and Senescent Cells

**Figure 4:**
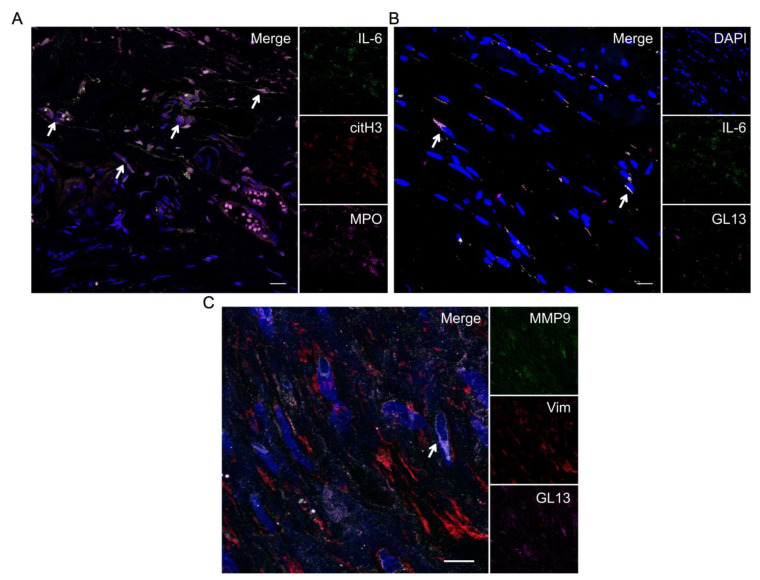
(**A**) Representative picture of IL-6+ NETs in the TABs from GCA patients [151]. White arrows indicate the co-staining of MPO/citH3/IL-6. (**B**) Representative picture of senescent cells positive for IL-6 in the TABs from GCA. White arrows indicate the co-staining of GL13/IL-6. (**C**) Representative picture of senescent cells positive for MMP-9 in the TABs from GCA [188]. White arrows indicate co-staining of MMP-9/GL13. Blue: DAPI; green: (**A**) + (**B**) IL-6, (**C**) MMP9; red: (**A**) citrullinated H3, (**C**) vimentin; magenta: (**A**) MPO, (**B**) + (**C**) GL13/lipofuscin. Objective (**A**) + (**B**) 20×, (**C**) 40×. Scale bar: 25 μm.

In recent studies, the properties of two cellular structures in tissue injury of GCA have emerged. These are neutrophil extracellular traps (NETs) and senescent cells, which we have recently detected in GCA TABs and have been associated with IL-6 expression [151,188] (Figure 4).

Netosis is a lytic type of cell death that is activated upon neutrophils’ encounter with pathogens as seen during infection, but also under sterile inflammation [189]. A characteristic disassembly of neutrophil cytoskeleton, histone citrullination, chromatin decondensation, and assembly of toxic granules containing myeloperoxidase (MPO) and neutrophil elastase (NE) are the hallmarks of this mechanism [190]. There is increased scientific interest in the organ-specific effects of NETs in autoimmune diseases such as systemic lupus erythematosus (SLE), ANCA vasculitis, and psoriasis [191,192,193]. The protein decoration of NETs (MPO, proteinase 3) causes the maintenance of the inflammatory response via autoantibody generation in an ANCA-associated vasculitis mouse model [194]. NETs also serve as a scaffold for thrombus build-up and platelet aggregation in patients with antiphospholipid syndrome (APS) [195]. In this direction, our group has reported the presence of NETs decorated with IL-6 and IL-17A in TABs from GCA patients [151]. However, the role of these toxic structures requires further clarification in the disease’s pathogenesis.

Cellular senescence is a phenomenon in which normal cells, upon intrinsic and extrinsic stimuli, are led to cell cycle arrest followed by metabolism dysregulation, macromolecular damage, and a specific senescence-associated secretory phenotype (SASP) [196]. The SASP secretome includes a variety of proinflammatory mediators, chemokines, growth factors, angiogenic factors, bioactive lipids, and MMPs that may act in a paracrine or autocrine manner to induce senescence in vivo, triggering a positive-feedback loop that leads to the perpetuation of the inflammatory response [197]. Senescent cells have been identified in the context of human diseases, including COVID-19 [198], where senescent cells were detected in SARS-CoV-2-infected lung tissue, and Hodgkin lymphoma, where a high percentage of senescent cells were detected in the affected lymph nodes [199,200]. Our group has reported the presence of senescent cells in biopsy-proven GCA specimens [188]. Senescent cells in GCA tissue mainly originate from fibroblasts, endothelial cells, and macrophages, while the detected SASP involves IL-6 (fibroblasts and macrophages) and MMP-9, with the latter restricted to senescent fibroblasts. CGA artery culture-conditioned media induced IL-6-associated senescence in primary fibroblasts in vitro, an effect that was reduced upon successful IL-6 signaling inhibition by tocilizumab. Another recent study has also reported the implication of activated senescent pathways in the pathogenesis of GCA [201]. However, the presence of senescent cells was not illustrated since their approach did not involve the consensus guideline multimarker algorithm adopted by the International Cell Senescence Association [196,202].

Interestingly, these two structures are potentially linked. A recent study addressing tissue remodeling mechanisms in the context of retinopathies demonstrated that senescent cells, via their SASP components, attract neutrophils, which release NETs and ultimately clear senescent cells, contributing to tissue remodeling [203].

**Figure 5:**
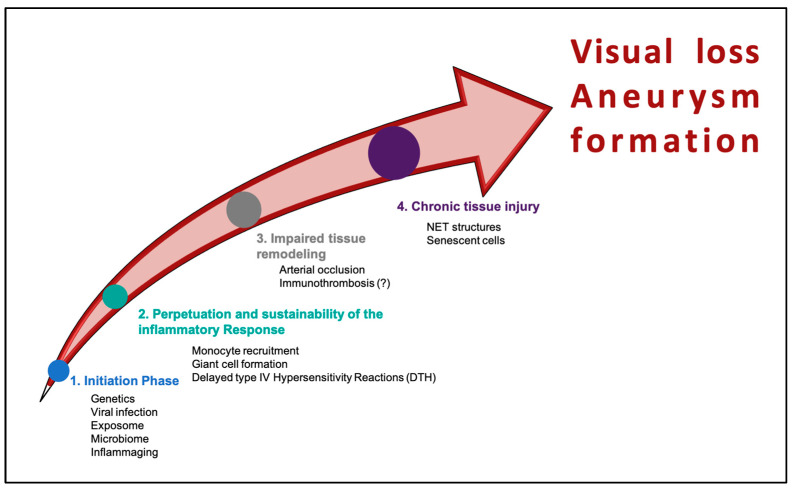
Phases of inflammation and tissue injury in giant cell arteritis presented as sequential events. In most cases, these phases overlap.

NETs and senescent cells share common properties that may affect the sustainability of autoimmune response to tissue injury. Indeed, they may stay for long periods in inflamed or (probably most importantly) in post-inflamed tissue. They are detected in GCA-affected tissue decorated with inflammatory cytokines and/or MMPs, thus affecting tissue remodeling. Further studies are required to address the function of these inflammatory structures in detail and to decipher whether they contribute to the perpetuation of the inflammatory response or chronic tissue injury detected in GCA (Figure 5).

### 3.2. Lipid Mediators in Vascular Inflammation

Unresolved inflammation is crucial for the pathophysiology of commonly occurring vascular diseases such as atherosclerosis and aneurysm formation [204]. Despite being an immune-driven vascular disease, research on GCA pathogenesis has consistently neglected and never investigated mechanisms of the resolution of inflammation, mediated by ω6 and ω3 polyunsaturated fatty acids (PUFAs), critical regulators of inflammation [205]. It is well established that within self-limited acute inflammatory exudates, pro-resolving families of lipid mediators are identified, termed resolvins, protectins, and maresins, which actively stimulate cardinal signals of resolution, including cessation of leukocytic infiltration, counter-regulation of proinflammatory mediators, and uptake of apoptotic neutrophils and cellular debris [205]. The biosynthesis of these resolution-phase mediators in sensu stricto is initiated during lipid-mediator class switching, which is the biochemical transition from inflammation to resolution, wherein early proinflammatory lipid mediators (LMs) such as prostaglandins and leukotrienes are replaced by specialized pro-resolving lipid mediators (SPMs) in exudates. The mechanisms of this shift in the LMs profile involve temporal changes in the expression, localization, and activity of critical cellular enzymes such as 5- and 15-lipoxygenases (LOXs) [206]. Once available, SPMs coordinate crosstalk between leukocytes and local cell populations, promoting an M1–M2 phenotypic transition in macrophages that is central to tissue repair [207]. SPMs further promote resolution by positive-feedback effects on LOX activity and SPM receptor expression in leukocytes [208]. Translation of these concepts into therapies for complex human diseases is the current challenge faced by investigators in the field of inflammation.

Evidence suggests that the resolution of vascular inflammation is an essential driver of vessel wall remodeling and functional recovery [204]. According to recent studies on vascular inflammation, stromal cells and their interactions with leukocytes are directly impacted by SPMs, which are locally synthesized in vascular tissues and have protective effects on the injury response [204]. Moreover, studies imply that the ratio of proinflammatory LMs to SPMs may reflect a systemic indicator of poor prognosis, greater tissue damage, and high mortality [209,210]. For this reason, measuring SPMs and proinflammatory LMs in both TABs and the periphery of GCA patients is of the greatest importance.

Studies of lipid mediators in GCA are lacking, and this is a significant unmet need for the disease. A speculation that urgently needs to be proven is that GCA patients might have an imbalance of proinflammatory LM/SPMs with a predominance of a “hazardous” LM signature that exacerbates hyperinflammation and tissue damage. Research is required to identify the potential for “resolution therapeutics” in GCA, enhance clinical measurement tools, and comprehend the molecular and cellular mechanisms of resolution in the vasculature.

## 4. Experimental Models in GCA

### 4.1. Mouse Models in GCA

While no specific mouse model perfectly mimics all aspects of GCA, researchers have developed a few experimental models to study different aspects of vasculitis [211]. Of the proposed animal models in GCA, the most widely used is the human artery xenograft mouse model, in which human artery implantation is established in immunodeficient mice [185,212]. It is the only model that has been useful to test various therapeutics for GCA (Table 2). Although the use of immunodeficient mice provides a simulation of the human environment in vivo, several challenges persist, including, among others, the increase in the survival rate of mice to extend the observation duration and the efficient reconstruction of the human B-cell immune response [211].

As TLRs have been implicated in the pathogenesis of LVV [213], activating adventitial DCs either in the vasa vasorum area or in perivascular adipose tissue is another method to induce arteritis in mice. However, this method is technically challenging, particularly in rodents, due to the thin artery width and the questionable presence of vasa vasorum in the mouse aorta [214,215] (Table 2). Another effort for the development of vasculitis in mice is the induction of arterial injury through arterial cuffing in prone models for arteritis or through an intraluminal balloon (coupled with TLR ligands). More modified arteritis-prone models have shown encouraging data for establishing an LVV model [216,217,218].

It is important to note that while these models exhibit certain features of vasculitis, none fully replicate the complexity and heterogeneity of human LVV, while others have not been investigated in the context of GCA pathogenesis yet (Table 2). Researchers often use a combination of these models and study various aspects to gain insights into the pathogenesis, underlying mechanisms, and potential therapeutic interventions for large vessel vasculitis in humans. Additionally, advances in modeling techniques continue to refine these models for better representation and understanding of systemic autoimmune vasculitis.

**Table 2:** Mouse models in the research of GCA pathogenesis.

Animal Model	Mechanism of Action	Limitations	Ref, Year
Human artery engraft	in NOD-SCID mice	leakage, spontaneous thymomas, very short lifespans	[212], 1997 [185], 2017
Induction of arteritis through administration of TLRs and activation of adventitial DCs	at the vasa vasorum	technically challenging for rodents	[214], 2022 [215], 2003
	at perivascular adipose tissue (PVAT)	involvement in atherosclerosis, speculation for GCA pathogenesis	[219], 2022 [220], 2000 [221], 2005
Induction of arterial injury through arterial cuffing	in genetically modified atherosclerosis-prone mice	speculation for GCA pathogenesis	[216], 2002
	in IBP-deficient or IL-1Ra deficient mice	not sufficient to initiate pan-arteritis	[217], 2003 [218], 2014
Induction of arterial injury through intraluminal balloon	in atherosclerotic mice	tested in mouse and rat models of atherosclerosis, speculation for GCA pathogenesis	[222], 2007
Modified arteritis-prone models	IL1-A deficiency mice upon artery injury	arteritis development is dependent on the genetic background	[223], 2005
	or IL25 administration	speculation for GCA pathogenesis	[224], 2019
	IBP-deficient mice with TCR specific to OVA peptides	speculation for GCA pathogenesis	[225], 2008
Infection-related chronic vasculitis	IFNg-receptor-deficient mice infected with γ-herpes virus 68	IFNg signaling deficiency not investigated in LVV patients	[226], 2001 [227], 1998

### 4.2. Current and Future In Vitro Models of LVV

Artery culture models: For the last decade, most groups have depended on using in vitro models of whole temporal artery culture to address questions requiring functional studies. In this context, two models have been proposed in the literature. The first and the most widely used model involves small temporal artery sections cultured in a tri-dimensional matrix for five days that facilitates the study of protein and RNA kinetics after specific drug treatments [109]. This model has been successfully used to investigate the effect of significant cytokines in disease pathogenesis [147,176]. However, it involves some limitations, including a bias in the levels of different molecules originating from the use of Matrigel and the ex vivo manipulations of the model. The second proposed in vitro model involves the culture of temporal artery sections for a shorter period (24 h) without using any supporting matrix [228]. This model is implemented to collect the secreted inflammatory microenvironment of a GCA-affected artery and use it as a conditioned medium for further applications (e.g., in treating PBMCs, endothelial cells, and myofibroblasts) [229].

Even though these models have expanded our understanding of GCA pathogenesis, they do not facilitate the investigation of the early phases of disease initiation or chronic tissue remodeling. With the advancements in the diagnostic approaches in GCA and when temporal artery biopsy for diagnostic purposes will be obsolete, establishing such models will not be feasible. Thus, new in vitro models are required for functional studies in this field.

Bioengineered arteries: Bioengineered blood vessels have attracted the scientific community’s interest in the last decade in an attempt to establish in vitro models for studying cardiovascular diseases and reducing the use of laboratory animals [230]. Along this line, several vasculature models have been suggested for constructing vessels of different sizes. A bioartificial artery model for large arteries has been described, implementing a collagen type I matrix that served as a scaffold for VSMC and EC lining [231]. The constructed bioartificial arteries were cultured for two days and were populated with DCs, monocytes, and macrophages to mimic the human vasculature in the initiating events of vasculitis. Even though the construct closely resembled human medium-sized arteries, the model exhibited several limitations, including the short period of viability, lack of patient-derived vascular cells, and the lack of following culture protocols underflow to mimic human contractile vasculature [231]. Another proposed model for the construction of human arteries incorporating 3D bioprinting approaches was described by Xu et al. This novel approach successfully produced vessels that closely mimic human medium-sized vessels but did not involve using immune cells to test this methodology in the setting of autoimmune disease research [232]. Moreover, patient-derived induced pluripotent stem cells are strongly suggested in constructing 3D in vitro models to retain the epigenetic, transcriptomic, and metabolic profile of the patient of origin [233,234]. The abovementioned efforts to construct bioengineered human arteries indicate that the ever-expanding tools and novel methodologies hold the potential for the generation of more physiologically relevant in vitro models of large vessel vasculitis in the foreseeable future.

## 5. Conclusions

GCA constitutes an ideal model to study acute and chronic DTH responses. In this review, we described, for academic purposes, the continuous inflammatory process as four distinct phases: (i) the initiation of the disease; (ii) the perpetuation and sustainability of the inflammatory response; (iii) the impaired tissue remodeling; and (iv) the chronic tissue injury phase (Figure 5). Despite the enormous progress over the past few years in understanding disease mechanisms, diagnosis, and application of targeted therapies, many unanswered questions still need to be answered. New queries have been generated, leading eventually to new unmet needs for the disease and, therefore, a revised research agenda. Among them, the generation of accurate animal models of the disease or the use of bioengineered artery models for obtaining more insights into pathogenetic mechanisms, the application of different omics technologies, and the integration of generated data in artificial intelligence-empowered platforms in pairs of samples during activity and inactivity of the disease, will undoubtedly provide new biomarkers with direct mechanistic involvement and composite characteristics. New treatment targets will be identified, and revised treatment selection tools will be developed, taking into consideration patient characteristics and future comorbidities.
